# Impaired Macrophage and Satellite Cell Infiltration Occurs in a Muscle-Specific Fashion Following Injury in Diabetic Skeletal Muscle

**DOI:** 10.1371/journal.pone.0070971

**Published:** 2013-08-12

**Authors:** Matthew P. Krause, Dhuha Al-Sajee, Donna M. D’Souza, Irena A. Rebalka, Jasmin Moradi, Michael C. Riddell, Thomas J. Hawke

**Affiliations:** 1 Department of Pathology & Molecular Medicine, McMaster University, Hamilton, Ontario, Canada; 2 Muscle Health Research Centre, York University, Toronto, Ontario, Canada; University of Minnesota Medical School, United States of America

## Abstract

**Background:**

Systemic elevations in PAI-1 suppress the fibrinolytic pathway leading to poor collagen remodelling and delayed regeneration of tibialis anterior (TA) muscles in type-1 diabetic Akita mice. However, how impaired collagen remodelling was specifically attenuating regeneration in Akita mice remained unknown. Furthermore, given intrinsic differences between muscle groups, it was unclear if the reparative responses between muscle groups were different.

**Principal Findings:**

Here we reveal that diabetic Akita muscles display differential regenerative responses with the TA and gastrocnemius muscles exhibiting reduced regenerating myofiber area compared to wild-type mice, while soleus muscles displayed no difference between animal groups following injury. Collagen levels in TA and gastrocnemius, but not soleus, were significantly increased post-injury versus controls. At 5 days post-injury, when degenerating/necrotic regions were present in both animal groups, Akita TA and gastrocnemius muscles displayed reduced macrophage and satellite cell infiltration and poor myofiber formation. By 10 days post-injury, necrotic regions were absent in wild-type TA but persisted in Akita TA. In contrast, Akita soleus exhibited no impairment in any of these measures compared to wild-type soleus. In an effort to define how impaired collagen turnover was attenuating regeneration in Akita TA, a PAI-1 inhibitor (PAI-039) was orally administered to Akita mice following cardiotoxin injury. PAI-039 administration promoted macrophage and satellite cell infiltration into necrotic areas of the TA and gastrocnemius. Importantly, soleus muscles exhibit the highest inducible expression of MMP-9 following injury, providing a mechanism for normative collagen degradation and injury recovery in this muscle despite systemically elevated PAI-1.

**Conclusions:**

Our findings suggest the mechanism underlying how impaired collagen remodelling in type-1 diabetes results in delayed regeneration is an impairment in macrophage infiltration and satellite cell recruitment to degenerating areas; a phenomena that occurs differentially between muscle groups.

## Introduction

Type 1 diabetes mellitus (T1DM) is an autoimmune disease defined by hyperglycemia and hypoinsulinemia in the absence of exogenous insulin treatment. Unfortunately, exogenous insulin alone does not represent a cure, but rather a therapy that fails to completely normalize the widespread disturbances in growth and energy metabolism. In the absence of a true cure, it is the complications of diabetes that define the overall health of the affected person. One such complication that has recently received attention is diabetic myopathy [1]–[4]. The pathophysiology of diabetic myopathy includes impaired muscle growth, as well as immediate and progressive loss of muscle mass and contractile function [1], [2], [4]–[8]. The evidence to date suggests this is, in part, due to an imbalance between protein synthesis and proteolysis in the diabetic muscle [9]–[11] and is observed as a reduction in muscle mass and myofiber cross-sectional area. While the above situation is concerning in terms of muscle growth, another series of complications arise in response to muscle overuse or to muscle injury, where a multifaceted regenerative process is needed to repair/replace damaged myofibers. In this case, a severe impairment in muscle repair is observed in the skeletal muscle of T1DM; a distinct problem that is not related to any imbalances in protein metabolism [2], [12]. From a clinical perspective, as increased physical activity is now recognized as a critical therapeutic tool for most T1DM patients [13], defining the capacity (and underlying mechanisms for impairments) of skeletal muscle to grow, adapt and repair from exercise and other stressors in diabetes becomes of paramount importance.

The repair of skeletal muscle is a complex orchestration of events including infiltration of immune and muscle stem cells (also termed satellite cells), degeneration or repair of damaged myofibers, extracellular matrix (ECM) remodelling, and formation and subsequent growth of new myofibers [14]. The activity of plasminogen activators (PA), such as urokinase PA (uPA), are requisite for skeletal muscle regeneration [15]–[19], but PA activity is limited by plasminogen activator inhibitor-1 (PAI-1), a diurnally regulated hormone that is significantly and chronically elevated in both type 1 and type 2 diabetes [2], [20]–[22]. Recently, our lab demonstrated PAI-1 as a mediator of the delayed muscle regeneration in the Akita mouse model of T1DM. Pharmacological reductions in PAI-1 activity (through PAI-039) reduced collagen levels and restored muscle regeneration [2]. PAI-039 treatment increased active uPA and matrix metalloproteinase-9 (MMP-9) levels in Akita mouse tibialis anterior (TA) muscles, supporting the hypothesis that PAI-1 suppresses the proteolytic activity of uPA and MMP-9 in diabetes, thereby impairing the regenerative process [2].

Though we, and others, have shown that PAI-1 suppresses uPA activity, and ultimately muscle regeneration [2], [19], the specific mechanism(s) underlying how impaired collagen degradation was delaying regeneration remained unanswered. We hypothesized that excessive collagen levels in the muscle of Akita mice would attenuate infiltration of macrophages and satellite cells into the damaged areas leading to a delay in muscle regeneration. Furthermore, in rodent studies of T1DM, muscle groups with a high proportion of glycolytic myofibers [such as the TA, gastrocnemius, and extensor digitorum longus] appear to suffer the greatest myofiber atrophy, while the soleus consistently demonstrates resilience to diabetic myopathy [4], [6]–[8], [23]–[26]. Thus, we further hypothesized that there would be muscle group-specific differences observed in terms of inflammatory and satellite cell infiltration and regenerative capacity with the resilience of the soleus extending to the process of muscle repair. Our findings support the hypothesis that PAI-1 mediated delay in ECM remodelling leads to a delay in macrophage and satellite cell infiltration into the damaged areas of diabetic skeletal muscle. We also demonstrate that although the increase in PAI-1 is systemic, intrinsically higher inducible expression of MMP-9 in response to injury within the oxidative, postural muscles (soleus) versus more glycolytic, non-postural muscles (TA) provides a mechanism for the soleus (and other oxidative) muscles to maintain normal ECM turnover during injury and ultimately maintain normal regenerative capacity.

## Methods

### Animal Care

All animal experiments were approved by the McMaster and York University Animal Care Committees in accordance with Canadian Council for Animal Care guidelines.

Male C57BL/6-*Ins2* ^Akita^/J (hereafter referred to as Akita) mice and their wild-type littermates (WT) were purchased at 3 weeks of age from Jackson Laboratory (Bar Harbor, ME). Akita mice and WT mice (n = 12) were studied over a period of up to 8 to 13 weeks of untreated T1DM (depending on time-point of regeneration measured). Akita mice become spontaneously diabetic at ∼4 weeks of age due to a heterozygous mutation in the *Ins-2* gene. Monitoring of diabetes onset was performed as described previously [2], [4].

To determine if restoration of PAI-1 activity in the diabetic mouse would normalize macrophage infiltration into the regenerating TA, an additional group of WT and Akita mice (n = 4) were treated via oral gavage with vehicle (2% Tween-80 and 0.5% methylcellulose in sterile H_2_O) or vehicle plus PAI-039 (2 mg/kg; Axon Medchem, Netherlands, axon1383), an orally effective inhibitor of active PAI-1 [27], [28]. On the day of CTX injury (at 8 weeks of diabetes, or approximately 12 weeks of age), the mice were treated with vehicle or vehicle plus PAI-039 at 1100 hours, then received CTX injury to the TAs with 10 uM CTX at 1200 hours, and received PAI-039 treatment again at 1500 hours. PAI-039 treatment was continued twice daily at those designated times throughout the 5 day regeneration period, at which point the animals were sacrificed and tissues were dissected and stored as described below. Those treatment time-points were chosen to best attenuate the peak of PAI-1 activity due to its circadian expression pattern. As well, as collagen levels within the resting skeletal muscle of these young adult Akita and WT mice were not different (not shown), administration of PAI-039 began 1 hour before injury as there was not a need to act on the established extracellular matrix. WT mice treated with vehicle (WT+vehicle) demonstrated no significant difference from untreated WT in any variable measured and these groups were pooled for comparison with Akita mice and Akita mice treated with PAI-039 (Akita+PAI-039).

Blood glucose and body mass were measured biweekly with animals in the fed state. Blood samples were collected at 6 weeks of diabetes via tail-nick for analysis of metabolites, hormones and other cytokines. The animal room was maintained at 21°C, 50% humidity and 12 h/12 h light-dark cycle. All mice had access to standard breeder chow, water ad libitum and enrichment material.

### Skeletal Muscle Injury and Tissue Collection

Skeletal muscle injury was induced following 8 weeks of diabetes (approximately 12–13 weeks old) via an intramuscular injection of 100 µL of 10 µM cardiotoxin (CTX; Latoxan, Valence, France) per muscle, as previously described [29]. Injuries were generated in the left TA and gastrocnemius-plantaris-soleus (GPS) muscles of both Akita and WT mice. These mice were divided into 3 groups to allow different lengths of recovery (5, 10, or 35 days post injury). Following the specified regeneration period, animals were euthanized by CO_2_ inhalation and cervical dislocation, and tissues were collected and stored appropriately for future analyses. When muscles were dissected, both CTX-treated and untreated TA and GPS muscle groups were weighed for comparison.

### Blood Analyses

Heparinized blood plasma was analyzed for insulin and total PAI-1 (Multiplex Adipokine assay from Millipore, MADPK-71K) at all collection time points. Plasma was also analyzed for non-esterified fatty acids (NEFA) with the use of a colorimetric assay (NEFA-HR2, Wako diagnostics, Wako TX).

### Histochemical and Immunofluorescent Analyses

Frozen OCT-embedded skeletal muscle was cut using a cryotome into 8 µm cross-sections and mounted on untreated glass slides for histochemical or immunofluorescent staining.

#### H&E staining

Hematoxylin and eosin staining was used to determine average cross-sectional area of uninjured and injured myofibers. Three images spaced evenly throughout the TA were used for analysis where 25 fibers per image were analysed for mean fiber area (75 total fibers per TA). In the soleus and gastrocnemius, between 50–75 fibers were analysed to represent fiber area, as has previously been validated [30], [31].

#### Picrosirius red staining

To stain for collagen content, picrosirius red staining was performed as previously described [2], [32]. The myofibers appear yellow while the collagen retains the red stain, providing adequate contrast for collagen [2].

#### Immunofluorescent Staining

Muscle section preparation was performed as previously described [2]. To identify Myh3-expressing myofibers, sections were incubated with 1∶1 dilution of anti-Myh3 (F1.652; DSHB, Iowa City, IO) in blocking buffer 1.5% normal goat serum and 1.5% horse serum albumin) overnight at 4°C. Similarly, F4/80 antigen was used to identify macrophages in injured muscles, using a dilution of 1∶200 F4/80 antibody (AbD Serotec, CedarLane Labs, Burlington, ON), however, mouse IgG block was omitted. Appropriate secondary antibody application was performed (Alexa 488 anti-mouse antibody [Invitrogen, A-11001] or Alexa 594 anti-rat antibody [Invitrogen, A-11007]) followed by 5 min of 1∶1000 4,6-diamidino-2-phenylindole (DAPI) to identify nuclei. To facilitate identification of positively stained myofibers or macrophages, either laminin or type I collagen was stained for using anti-laminin (ab14055, Abcam, Cambridge, MA) or anti-collagen, type I (ab292, Abcam). Satellite cells were identified by immunostaining for Pax7. Freshly cut 5 µm sections were air-dried, then fixed for 4 minutes in ice-cold 2% PFA. Sections were then incubated in 1% triton X-100 for 30 minutes followed by blocking buffer for 30 minutes. Pax7 antibody (DSHB) was diluted 1∶1 in a blocking buffer and incubated overnight at 4°C. A biotin-streptavidin (Vector Labs, Burlington, ON) detection system was used with Dylight 594 (Abcam, 1∶250 in blocking buffer) used to visualize Pax7 positive nuclei with a DAPI co-stain.

### Image Analysis

Images of stained sections were obtained with a Nikon 90i eclipse upright microscope (Melville, NY) and analyzed using Nikon Elements software. Analysis included determination of collagen, Myh3, and F4/80 positive area using signal threshold settings as the detection method. In all staining procedures, a negative control (no primary antibody) was performed and used to set the signal threshold settings. In all cases, negative controls displayed an absence of signal. For determination of F4/80 cell number, the threshold binary for DAPI-stained nuclei was intersected with the F4/80 threshold binary; the number of intersected objects represented the positive cell number. Regenerating (injured) and uninjured muscle fiber area in H&E stains was determined manually using Elements software. Both regenerating fibers, easily differentiated from uninjured fibers due to their small size with centrally-located nuclei, and uninjured fibers were manually encircled using the software thus identifying their cross-sectional area. TA, gastrocnemius, and soleus muscles were analyzed separately to determine muscle group-specific differences. Regenerating and necrotic injured muscle tissue represent different stages of the overall regeneration process, and, thus, were identified in immunofluorescently stained tissue and analyzed separately as specifically illustrated within our figures and similar to the analyses conducted previously [33].

### Immunoblotting

Homogenates of soleus, gastrocnemius and TA muscles from WT mice were prepared as before [2]. Fifteen micrograms of protein sample were loaded, resolved by 10% SDS-page gels, and transferred to PVDF membranes. Primary antibodies for total MMP-9, (1∶5000, Abcam) and beta-actin (1∶2500, Abcam) were used to detect proteins of interest, and were subsequently conjugated with the appropriate horseradish peroxidase secondary antibodies. Signals were visualized using chemiluminescent reagent (GE Healthcare, Baie d’Urfe QC), and acquired with the CareStream imager and accompanying software (Woodbridge, CT).

### Statistical Analysis

For all experiments, the appropriate t-test or two-way ANOVA with Bonferroni post-hoc analysis of pairwise comparisons was carried out to classify significant differences (P<0.05) between Akita and WT groups. Two-way ANOVA was run on data sets with dependent variables measured over time, while one-tailed t-tests were carried out on data with only single comparisons. Data are presented as mean±SEM. An * denotes a significant difference identified by t-test or Bonferonni post-hoc test in pairwise comparisons, while a significant main effect of diabetes or a significant interaction between diabetes and time are listed within figure legends.

## Results

Akita mice spontaneously developed hypoinsulinemia and hyperglycemia at ∼4 weeks of age, which was maintained throughout the experiments (Table 1), consistent with previous findings [2], [4], [34]. Akita mice also demonstrated hyperlipidemia, elevated PAI-1 and attenuated body mass accrual by 6 weeks of untreated diabetes (Table 1), consistent with observations of T1DM in humans [20], [34], [35] and diabetic rodents [2], [21], [22], [31], [36], [37].

**Table 1:** Characteristics of wild type and Akita diabetic mice.

Measure	Group	Time of diabetes	
		6 weeks	9–13 weeks
**Body Mass (g)**	**WT**	24.9 (0.4)	27.7 (0.1)
	**Akita**	22.9 (0.4)*	24.5 (0.1)*
**Insulin (pg/ml)**	**WT**	814 (106)	1650 (218)
	**Akita**	222 (56)*	209 (24)*
**Blood Glucose (mM)**	**WT**	9.1 (0.3)	8.7 (0.2)
	**Akita**	32.4 (0.7)*	32.2 (0.4)*
**NEFA (mM)**	**WT**	1.10 (0.06)	0.66 (0.04)
	**Akita**	2.14 (0.12)*	1.59 (0.12)*
**PAI-1 (pg/ml)**	**WT**	429.6 (112.0)	291 (33)
	**Akita**	1593.5 (134.3)*	960 (217)*
**TA Mass (mg)**	**WT**		50 (2)
	**Akita**		41 (2)*
**GPS Mass (mg)**	**WT**		177 (6)
	**Akita**		141 (3)*
**TA fiber area (µm^2^)**	**WT**		2285 (92)
	**Akita**		1851 (36)*
**GAS fiber area (µm2)**	**WT**		2314 (102)
	**Akita**		1926 (83)*
**SOL fiber area (µm2)**	**WT**		1154 (47)
	**Akita**		1094 (41)

**Figure 1:**
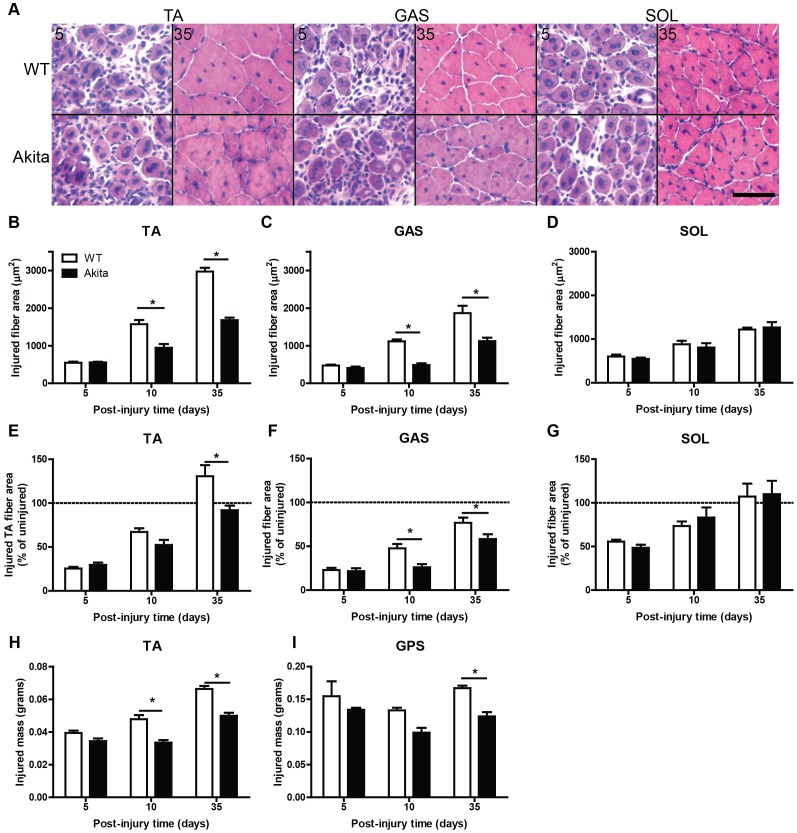
Soleus muscle is resilient to the regenerative defects found in other muscles from Akita mice. (A) H&E stained cryosections of tibialis anterior (TA), gastrocnemius (GAS), and soleus (SOL) illustrate decrements in muscle regeneration in diabetic Akita mouse muscles. (B) TA demonstrates the worst impairment in regeneration, as determined by cross sectional area of regenerating (centrally-nucleated) fibers (significant main effect of diabetes and interaction [P<0.05]). GAS (C) also demonstrates impaired regeneration (significant main effect of diabetes and interaction [P<0.05]), while SOL (D) does not. In panels E–F, TA (E), GAS (F), and SOL (G) CTX-injured fiber area data are expressed relative to fiber area from the contralateral uninjured muscles. The TA (significant main effect of diabetes and interaction [P<0.05]) and GAS (significant main effect of diabetes [P<0.05]) both demonstrate poor regeneration even when expressed in these relative terms. The mass of muscles that had been injured with cardiotoxin further illustrate poor regeneration. The muscle mass of the injured TA (H; significant main effect of diabetes and interaction [P<0.05]) and gastrocnemius-plantaris-soleus (GPS) complex (I; significant main effect of diabetes [P<0.05]) are illustrated here. * denotes significant post-hoc analysis differences (P<0.05). Scale bar represents 50 um.

Data collected at 6 weeks of diabetes (6 weeks group), or at 5, 10, or 35 days following cardiotoxin muscle injury at 8 weeks of diabetes (9–13 weeks group). All measures except for soleus fiber area were found to be significantly altered in the diabetic mice. All muscle mass and fiber area measures presented here are from the uninjured muscle; the cardiotoxin injured contralateral leg muscle data are presented in Figure 1. NEFA, non-esterified fatty acids; PAI-1, plasminogen activator inhibitor-1; TA, tibialis anterior; GPS, gastrocnemius-plantaris-soleus complex; GAS, gastrocnemius; SOL, soleus. * denotes significant difference in Akita compared to matching wild type (WT) value as assessed by t-test (P<0.05).

Assessment of the uninjured leg of Akita mice revealed significantly less muscle mass in the TA and GPS compared to WT (Table 1). Furthermore, mean myofiber area was reduced in Akita mice compared to WT in H&E stained cross-sections of TA and gastrocnemius. However, uninjured soleus of Akita mice exhibited no difference with WT in myofiber area (Table 1), consistent with previous studies [6]–[8], [23], [24]. Though soleus muscles were not weighed separately, given no difference in fiber area, we would hypothesize no difference between groups in soleus mass.

In response to cardiotoxin injury, Akita TA and gastrocnemius exhibited a significant loss of myofiber area in regenerating muscles compared to WT (Figure 1A–C), even when expressed relative to uninjured fiber area from the contralateral TA and gastrocnemius, respectively (Figure 1E, F). Conversely, soleus did not demonstrate a reduction in regenerating fiber area, in absolute or relative terms (Figure 1D, G). Masses of the injured TA and GPS complex were reduced in Akita mice compared to WT as well (Figure 1H, I), corresponding with the reductions in myofiber areas observed in the gastrocnemius and TA muscles.

**Figure 2:**
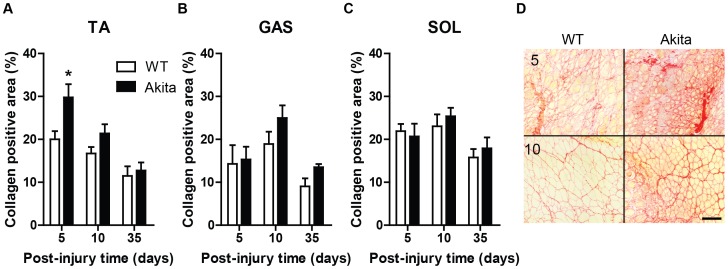
Tibialis anterior muscle of Akita mice demonstrate transient, excessive fibrosis during early regeneration. (A) TA demonstrates elevated collagen during regeneration, particularly at 5 days post-injury (significant main effect of diabetes [P<0.05]), while gastrocnemius (GAS) (B) demonstrates a non-significant trend (main effect of diabetes [P = 0.11]). Conversely, soleus (SOL) (C) does not demonstrate dysregulated collagen expression. * denotes significant post-hoc analysis differences (p<0.05). (D) Representative picrosirius red staining of 5 and 10 day post-injury TA muscles. Note the elevated presence of collagen (red) and smaller size of myofibers (yellow) in Akita TA. Scale bar represents 50 um.

Given this muscle-specific diversity in responses to the diabetic environment, further investigation into the regeneration process of Akita diabetic muscle was needed. It is known that ECM remodelling is a critical component of the regeneration process [2], [19], [31], [38], thus, we determined the expression levels of a primary ECM component, collagen. The injured TA muscles of Akita mice were found to have elevated collagen expression compared to WT, particularly at 5 days post-injury, as demonstrated by picrosirius red staining (Figure 2A, D). While the regenerating gastrocnemius exhibits a non-significant trend (P = 0.11), the regenerating soleus (P = 0.51) muscle does not demonstrate significant differences in collagen expression between Akita and WT mice (Figure 2B–C). The picrosirius red stained TA muscles also clearly demonstrate smaller fiber areas (yellow staining) observable at 10 days post-injury (Figure 2D), consistent with the analysis of H&E stained tissue sections.

**Figure 3:**
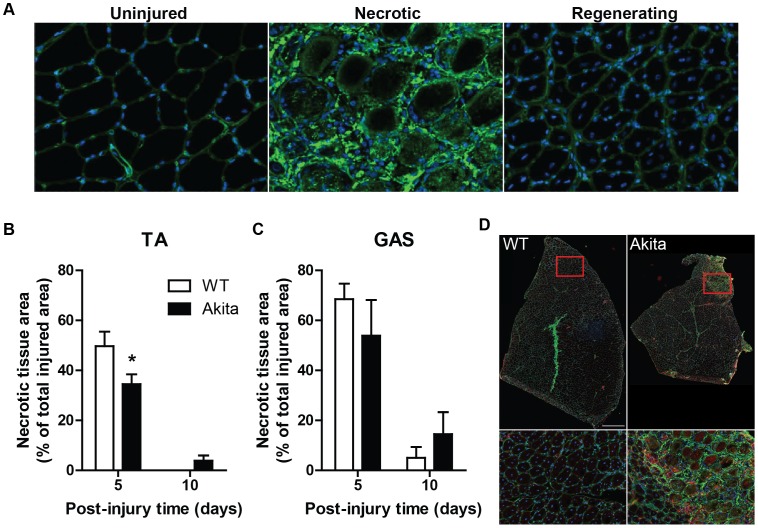
Necrosis of muscle fibers persists throughout muscle regeneration in Akita tibialis anterior muscle. (A) Uninjured (left), necrotic (center), and actively regenerating (right) regions of skeletal muscle are easily identified in collagen type I immunostain (green) with DAPI (blue) counterstain. Note the presence of centrally located nuclei in muscle fibers in regenerating muscle, indicative of new myofiber formation. (B) TA of diabetic mice have clearly defined areas of necrosis remaining at 10 days post-injury (significant interaction [P<0.05]; * denotes post-hoc difference). Similarly, gastrocnemius (GAS) (C) follows this pattern although not statistically significant (P = 0.21). In contrast, both WT and Akita soleus (SOL) display no areas of necrosis at 5 or 10 days post-injury (not shown). (D) Representative image of TA muscle undergoing regeneration at 10 days post-injury in WT and Akita TA. Note the distinct area of necrosis in the Akita TA. Scale bar represents 500 um.

Though the increased expression of collagen during the early phase of regeneration is important to maintain muscle integrity as the myofibers regenerate, we speculated that excessive collagen levels, particularly in the Akita TA muscles would attenuate the normal regenerative process. More specifically, we hypothesized that excessive collagen levels would delay the degeneration phase; a hypothesis that was tested by evaluating the size of necrotic versus regenerating areas within regenerating Akita and WT muscles. Sections of TA and GPS were immunofluorescently stained for type I collagen and DAPI so as to accurately identify necrotic and regenerating areas in each muscle (Figure 3A). Areas with highly disrupted collagen surrounding large, rounded (hypercontracted) myofibers were identified as damaged muscle undergoing necrosis, while areas with small, centrally-nucleated myofibers represented areas of regeneration. In each muscle, the sum area of the necrotic regions was determined and expressed as a percentage of the total injured area (necrotic area+regenerating area) in the muscle. The soleus did not have any remaining necrotic regions at 5 days post-injury in WT or Akita mice, whereas the TA and gastrocnemius exhibited varying amounts of necrosis at 10 days post-injury (Figure 3B–D). The TA of WT mice demonstrated a larger necrotic region size at 5 days post-injury (P<0.05) compared to the Akita TA. Necrosis was absent in the TA muscle of WT mice at 10 days post-injury, but still present in Akita mice (significant interaction: P<0.05; Figure 3B). Though a temporal pattern similar to that observed in the Akita TA was observed in the gastrocnemius, it did not reach statistical significance (P = 0.21; Figure 3C). Quantification of necrotic region size in tissue sections (depicted in Figure 3D), was confirmed in other sections stained for laminin and DAPI, as well as by H&E staining (not shown).

**Figure 4:**
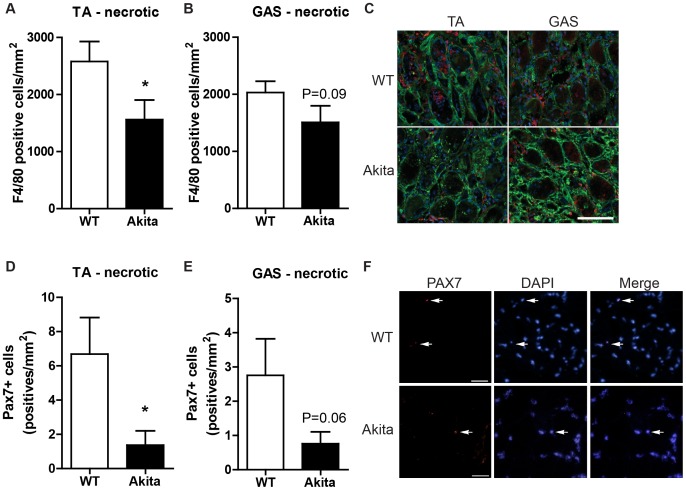
Macrophage and satellite cell infiltration into necrotic muscle is impaired in Akita diabetic mice at 5 days post injury. (A) The necrotic area of the TA demonstrates a reduced number of macrophages as evidenced by less F4/80 positive cells. * denotes significant difference by t-test (p<0.05). (B) A similar, though non-significant, trend of attenuated F4/80 positive cells was observed in necrotic gastrocnemius (GAS) (P = 0.09). (C) Representative images of necrotic regions of TA and GAS immunofluorescently stained for F4/80 (red), costained for type I collagen (green) and nuclei with DAPI (blue). Satellite cells (Pax7+ cells) were also reduced in number within necrotic areas of (D) Akita TA and (E) GAS (P = 0.06). (F) Representative images of Pax7 positive cells within necrotic regions of WT and Akita TA. Scale bar represents 50 um.

To determine if the impaired regenerative response observed in Akita TA and gastrocnemius muscles was a result of impaired macrophage infiltration secondary to elevated collagen levels, we undertook F4/80 immunostaining and quantification within the necrotic and regenerating regions of the injured muscles. In the Akita TA, a significant reduction in F4/80-positive cells was found in the necrotic region at 5 days post-injury (P<0.05; Figure 4A). A similar, but non-significant, trend (P = 0.09) was observed in the necrotic region of the gastrocnemius (Figure 4B). In necrotic areas, macrophages are typically found around the edge of the degenerating myofibers or entering the degenerating fibers as they are degraded and phagocytosed (Figure 4C). In regenerating regions of the injured muscles (TA, gastrocnemius, soleus) no significant difference between groups was noted in macrophage number at 5 or 10 days post-injury (Figure S1). Importantly, no difference in macrophage density was detected between groups in uninjured TA (WT: 643±25, Akita: 620±69 cells/mm^2^, P = 0.43), gastrocnemius (WT: 714±46, Akita: 659±64 cells/mm^2^, P = 0.26) or soleus (WT: 683±84, Akita: 810±38, P = 0.12) indicating that the observed impairment was in the regeneration process and not an extension of an inherent defect in diabetic mouse skeletal muscle or the basal, low-level, inflammatory state which characterizes diabetes.

Given the delay in macrophage infiltration into the necrotic regions of the TA and, to a lesser extent, the gastrocnemius of Akita mice, we speculated that satellite cell infiltration into the necrotic areas of damaged muscle may also be affected. For this analysis, we quantified the SC number per mm^2^ in the necrotic, regenerating and undamaged areas of all 3 muscle groups in the Akita and WT mice. Firstly, no significant defect in SC number was noted between Akita and WT mice, regardless of muscle, in the undamaged areas of the muscles (Table S1). Consistent with the deficit in macrophage infiltration, a significant decrease in satellite cell number within necrotic areas of TA was noted for Akita mice compared to control (Figure 4D), while the gastrocnemius exhibited a non-significant trend (P = 0.06). This impairment was not observed within the Akita soleus muscles due to the absence of necrosis at this time point. Also consistent with our F4/80 observations, no differences between Akita and WT was noted in SC density within regenerating areas of all 3 muscle groups (Table S1).

**Figure 5:**
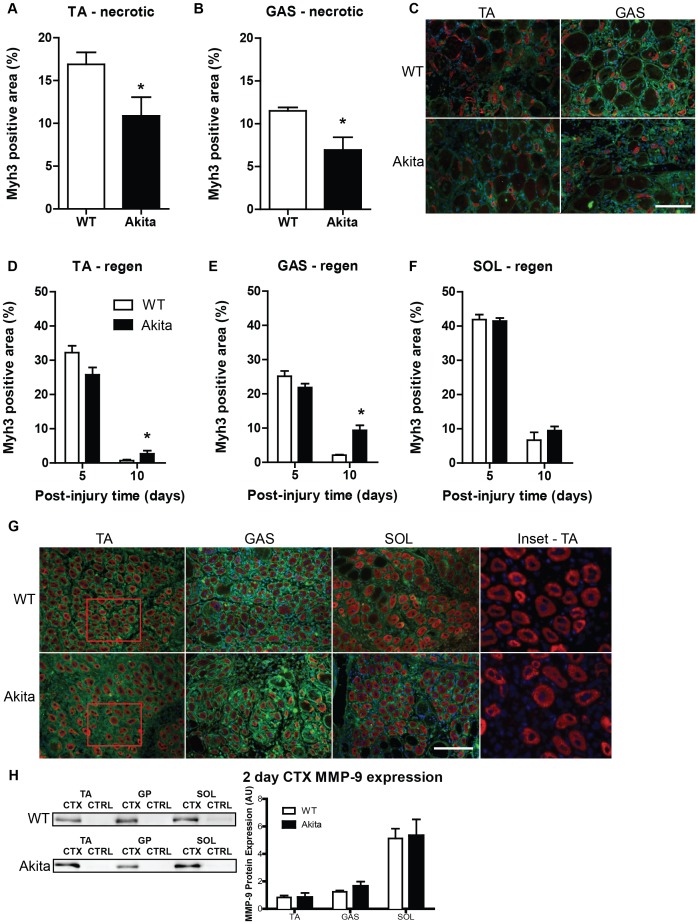
Initiation of muscle regeneration is delayed in tibialis anterior and gastrocnemius muscles in Akita mice. Regions of necrotic muscle tissue in (A) TA and (B) gastrocnemius (GAS) demonstrate attenuated response of Myh3-expressing myofibers in diabetic mice. An * denotes a significant difference detected by t-test (p<0.05). (C) Representative image of Myh3 immunofluorescent stained (red) TA and GAS necrotic regions. Co-staining of laminin (green) and nuclei with DAPI (blue) was performed. In the actively regenerating regions of the (D) TA and (E) GAS, the attenuation of Myh3 expression was not as severe, and at 10 days post-injury, was found to be significantly elevated in diabetic mice compared to wild type, indicative of delayed regeneration (significant interaction [P<0.05]). The * indicates significant differences detected by post-hoc testing (P<0.05). Conversely, (F) Akita soleus (SOL) demonstrates no impairment in establishing Myh3 expression following injury and does not continue to significantly overexpress Myh3 at 10 days post-injury. (G) Representative image of regenerating regions of muscles at 5 days post-injury stained for Myh3 (red) and laminin (green). Inset (derived from TA muscles) is provided without green channel to clearly demonstrate Myh3 stain morphology. Scale bar represents 50 um in panels C and G. (H) MMP-9 immunobloting on uninjured and regenerating (2 days post-injury) TA, GAS and SOL muscles from WT and Akita mice demonstrate the intrinisic differences between muscles in pro-MMP-9 expression (main effect of muscle group; P<0.05), but no differences between WT and Akita mice. MMP-9 content is relative to beta-actin loading controls and representative MMP-9 blots are included.

An important stage of skeletal muscle regeneration involves the de novo formation of myofibers from the fusion of myoblasts. These nascent myofibers will initially express the embryonic isoform of myosin heavy chain (Myh3) before expressing mature myosin isoforms. In the necrotic region of the TA at 5 days post injury, significantly less Myh3 expression was observed in Akita mice compared to WT (Figure 5A, C), as well as in the gastrocnemius (Figure 5B, C); observations that are consistent with the reduced infiltration of muscle satellite cells. In regenerating regions, expression of Myh3 observed at 5 days post-injury indicates a reduction in Akita mice (significant interaction: P<0.05). However, at 10 days post-injury, there is still significant expression of Myh3 in the Akita TA and gastrocnemius (Figure 5D–E) compared to WT (P<0.05), indicating attenuated repair as the regenerating WT muscles had already progressed to more mature myosin isoforms [39]. No delay in Myh3 expression was noted in the Akita soleus, consistent with no difference from WT in collagen levels or macrophage infiltration (Figure 5F). Size of Myh3-positive myofibers was also determined in the regenerating region of the three muscle groups and a similar pattern was observed. The gastrocnemius demonstrated a delay in the growth of the Myh3-positive cells (interaction: P<0.05), while the TA exhibited a trend for a delay (P = 0.078; Figure S2A–B). In contrast, the soleus did not demonstrate any delay in the growth of Myh3-positive myofibers (Figure S2C). These data again demonstrate a deficit in the regenerative capacity of Akita TA and gastrocnemius, but not soleus muscles.

While a PAI-1 mediated delay in collagen breakdown is a primary contributor to the attenuated muscle regeneration in TA muscles of Akita mice [2], we speculated that resilience of the soleus to the impaired regeneration in T1DM was the result of this muscle’s intrinsically greater inducible expression of MMP-9 [40]. Consistent with previous reports [40], minimal amounts of MMP-9 protein were observed in any of the uninjured skeletal muscles, and no difference was observed between Akita and WT. However, MMP-9 protein levels increased significantly post-injury, in a muscle-specific pattern, in both Akita and WT (main effect of muscle group: P<0.05; Figure 5H). It is also noteworthy that while soleus muscles exhibit the greatest inducible MMP-9 expression, a greater post-injury MMP-9 expression was observed in gastrocnemius compared to TA; a finding that might help explain the intermediate phenotype this muscle shows with respect to muscle regeneration in T1DM.

**Figure 6:**
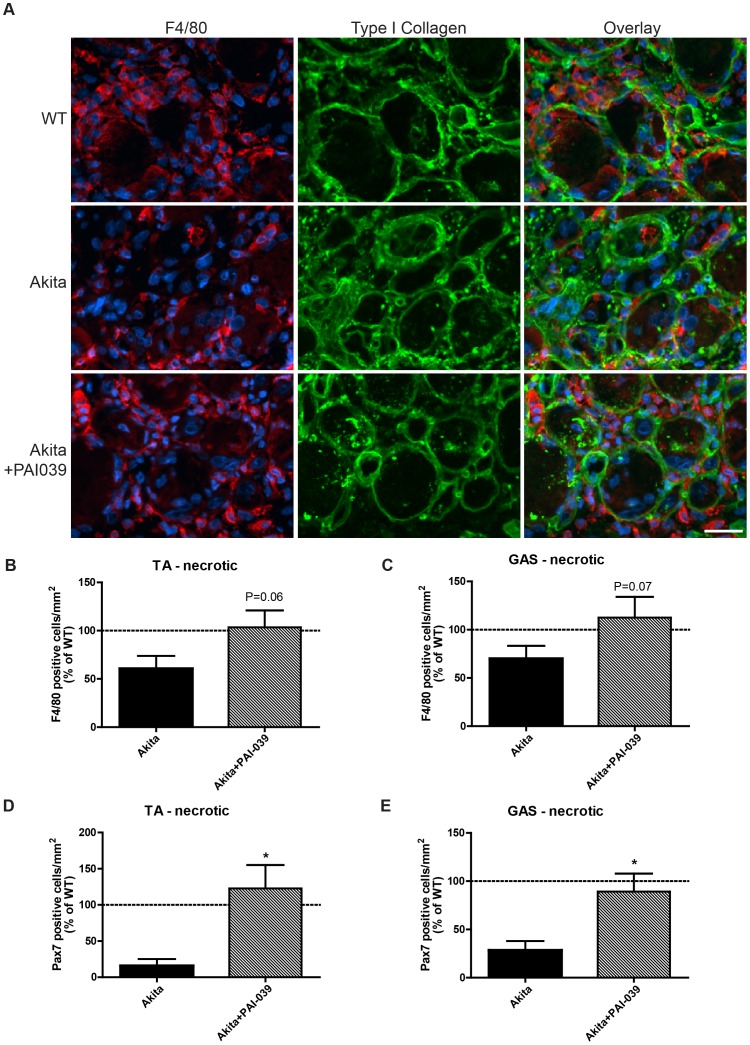
Macrophage and satellite cell infiltration into necrotic Akita diabetic muscle is restored with PAI-039 treatment. (A) Representative images of the necrotic area of the TA muscles of WT, Akita and Akita+PAI-039. Sections were stained with F4/80 (macrophage marker; red), collagen type 1 (green) and DAPI (nuclear dye; blue). A visible decrease in the number of F4/80 positive cells is seen in Akita muscle sections with a detectable increase in F4/80 cells in Akita TA muscles of mice treated with a PAI-1 inhibitor (PAI-039). (B) The number of F4/80 positive cells within the necrotic area of injured Akita TA muscles was returned to WT levels when these mice were treated with PAI-039. The number of F4/80 positive cells was greater in Akita+PAI-039 TA (panel B; P = 0.06) and gastrocnemius (panel C; P = 0.07) muscles compared to untreated Akita animals. A similar trend was observed for Pax7 positive cells which were also found to be improved in necrotic TA (D) and gastrocnemius (E) with PAI-039 treatment (P<0.05).

To support our hypothesis that the elevation in PAI-1 is the upstream mediator of the delayed infiltration of macrophages and satellite cells into the non-postural, more glycolytic muscles (TA, gastrocnemius) of Akita mice, we treated diabetic Akita mice with a PAI-1 inhibitor, PAI-039, during the regenerative process. PAI-039 administration returned F4/80 positive cell density in the necrotic areas of the TA muscles of Akita mice to that observed in WT mice, though this was not statistically significant (P = 0.06; Figure 6A, B). A similar trend was observed in the gastrocnemius: F4/80 cell density was improved but not significantly (P = 0.07; Figure 6C). Contrary to the deficit in Pax7 positive cells within the necrotic areas of TA muscle at 5 days post-injury (Figure 4D), PAI-039 treatment restored Pax7 positive cell number in the necrotic regions of the TA and gastrocnemius (P<0.05; Figure 6D, E).

## Discussion

Poorly controlled T1DM is associated with a number of negative effects on skeletal muscle including attenuated growth [2], [3], [4] and impaired regeneration [1]–[4], [12], [41]. We have demonstrated that the TA muscles of Akita mice exhibit impaired regeneration with as little as 7 days of diabetes exposure prior to injury [2]. Further, we defined the primary mechanism for this impairment as a chronic elevation of PAI-1 slowing ECM breakdown through reductions in uPA and active MMP-9 enzymatic activity. What remained unanswered was: (1) by what specific mechanism the attenuation of ECM turnover was delaying regeneration and (2) whether this phenomenon was occurring similarly in all muscle groups. This second point was most intriguing since it has been reported that the soleus muscle of streptozotocin-induced diabetic rodents is resistant to the negative effects of diabetes on growth and function [6]–[8], [23], [24] but it was not known if this extended to post-injury regeneration.

The findings of the present study reveal that exposure to the T1DM environment results in impaired skeletal muscle regeneration; a finding that is more pronounced in non-postural muscles with a greater glycolytic fiber-type composition. In the Akita TA, and to a lesser extent the gastrocnemius, muscle injury is followed by an excessive collagen accumulation (relative to WT). The present results suggest that this attenuation in collagen breakdown impairs the infiltration of macrophages and muscle satellite cells into the areas of damage and necrosis, ultimately prolonging the necrotic phase of muscle regeneration. Koh and colleagues [19] previously proposed that efficient removal of the ECM is necessary for effective macrophage and satellite cell migration during muscle regeneration and the current findings are in line with this hypothesis. Ultimately, this delay in degeneration translates into an attenuation of the regenerative phase, as illustrated by a temporal delay in the expression of developmental myosin heavy chain isoforms and reduced myofiber sizes. Interestingly, the soleus muscles of Akita mice did not display any of the impairments noted above, a finding of critical importance as we begin to unravel mechanisms underlying T1DM-mediated complications within skeletal muscle.

PAI-1 has been characterized as a primary suppressor of proteolytic activity in the extracellular space, slowing muscle regeneration down by inhibiting a cascade of protease activation. PAI-1 directly binds and inhibits uPA, the key plasminogen activator in muscle regeneration, preventing active plasmin from disrupting ECM proteins and from cleaving pro-MMP-2 and pro-MMP-9 into their active forms. These MMPs, particularly MMP-9, are crucial during the early stages of regeneration, functioning to enzymatically cleave protein chains composing the ECM; allowing for infiltration of both macrophages and satellite cells to regions of skeletal muscle damage [15]–[19], [42]. Indeed, the importance of MMP-9 to skeletal muscle ECM remodelling is demonstrated by our observations that Akita mice display reduced active MMP-9 expression [2] and that total MMP-9 inducible expression is greatest in soleus muscles compared to TA and gastrocnemius at 2 days following muscle damage (Figure 5). Such high levels are thought to promote rapid repair of the soleus, eliminating the necrotic area and excessive collagen expression that had been found in the glycolytic TA and gastrocnemius at 5 and 10 days following injury. It was hypothesized that intrinsically higher, inducible, MMP-9 expression would afford the soleus of Akita mice a mechanism by which this muscle is able to maintain normative regeneration rates. This prediction is based on reports of PAI-1 mediated fibrosis in cardiac and skeletal muscle [43], [44] and of a PAI-1 mediated-suppression of ECM remodelling (via uPA and MMP-9) as the primary contributor to delayed regeneration in the Akita TA [2].

While PAI-1 has been reported to impair macrophage infiltration via uPA inhibition within injured muscle [15], [17]–[19], [42], the fact that this is occurring in injured skeletal muscle of diabetic mice is a critical finding to unlocking the mechanisms underlying diabetic myopathy development. Macrophages play multiple roles in the regeneration process. Initially, these cells phagocytose damaged/dying muscle fibers and debris, but by 3–5 days post-injury, macrophages switch to a role of signalling for myoblast fusion and differentiation [45]. The present findings indicate that reduced macrophage infiltration in the diabetic mouse results in necrosis persistent at 10 days post-injury concurrent with delayed initiation of the regeneration phase indicated by decreased satellite cell infiltration and reduced Myh3 expression. Quite simply, these deficits in the degenerative and early regenerative phases could lead to attenuated muscle repair. These findings are consistent with the observations of others who have proposed that excessive collagen accumulation or impaired collagen turnover would attenuate macrophage and possibly satellite cell infiltration into the necrotic areas of damaged skeletal muscle [19]. Furthermore, our results demonstrating that systemic PAI-1 inhibition can restore the number of macrophages infiltrating the necrotic regions of injured TA and gastrocnemius muscles of diabetic mice to WT levels and ultimately restore the regenerative capacity lends further support to this hypothesis.

While we believe this cascade of events accurately depicts the deficits in Akita mouse TA muscle, differential effects were noted between muscles groups in these diabetic mice. Of the muscle groups studied in the current work, the TA demonstrated the most severe impairment in regeneration, while the soleus was clearly resistant to the diabetic environment with respect to both growth and regeneration. The gastrocnemius exhibited an intermediate phenotype, with impairments less severe than the TA, though growth and regeneration were still significantly delayed. This intermediate phenotype of the gastrocnemius is consistent with the mixed fiber-type distribution of this muscle. The evidence to date, derived from STZ-induced diabetic rodents, supports our findings that the soleus exhibits resilience against diabetic myopathy. Previous studies demonstrate that the soleus, which primarily expresses type I myosin heavy chain [46], is resistant to the atrophy or lack of growth induced by T1DM [6]–[8]. In fact, isolated type I fibers from diabetic rats demonstrate no loss of fiber size or contractile force [26], contrary to that found in muscles composed primarily of type II fibers [4], [7], [8], [23], [24]. Given previous [2] and present results, chronic elevations in PAI-1 appear to be the upstream mediator of the impaired regeneration observed in T1DM mice. Here we provide a mechanism as to how this rise in systemic PAI-1 could elicit such dramatic impairments within the TA but have little impact on the regenerative capacity of the soleus. The answer appears to lie with the intrinsic differences between these muscle groups in the fibrinolytic pathway. Indeed, during adolescent growth or following muscle injury, a greater inducible-expression and activity of MMP-9 is observed in oxidative muscle, such as the soleus, compared to the glycolytic muscle [40], [47]. As discussed, MMP-9 activity is critical for muscle regeneration to occur [38], [48], [49], and a greater inducible-expression in the diabetic soleus would allow repair to proceed normally, compared to the glycolytic TA and gastrocnemius, which lack the same degree of inducible MMP-9 expression. Evidence also exists that collagen types I, III and IV, the main constituents of skeletal muscle collagen, undergo a much milder gene expression response in the injured soleus compared to injured glycolytic muscles [50]. This implies that the soleus has a less extensive requirement for ECM remodelling compared to glycolytic muscle groups. Furthermore, uPA is highly expressed in soleus but not glycolytic muscle groups [51], meaning that despite increases in the PAI-1:uPA ratio, free (active) uPA may still be available in the diabetic soleus, affording it the opportunity to activate the proteolytic cascade. Thus, intrinsic differences between muscle groups, in terms of ability to remodel the ECM and the amount of remodelling needed, would account for the observed differential response to injury noted in diabetic muscle groups.

Though we have proposed a PAI-1 mediated impairment in collagen remodelling as the primary mechanism of attenuated regeneration in diabetic mice, an alternative scenario (which need not be mutually exclusive) could involve impairments in macrophage and satellite cell proliferative/migratory capacity in Akita mice. While this hypothesis was untested in the current study, our present results suggest that a global defect in satellite cells or macrophages is not likely the primary mediator of the impaired muscle regeneration as deficits in muscle repair were not exhibited in the soleus muscles of diabetic Akita mice. What’s more, no difference in the number of Pax7-positive cells was noted between groups in the regenerating soleus 5 days post injury. Finally, the ability of PAI-039 to restore normative regeneration (as assessed by collagen levels and Myh3 levels) [2] suggests that the plasminogen to MMP-9 pathway is the primary defective pathway in this diabetic model. Clearly, future studies are needed to further investigate the effects of the T1DM environment on satellite cell and macrophage functional capacities.

### Conclusions

In conclusion, our data support the novel hypothesis that skeletal muscle regeneration in T1DM is characterized by poor macrophage infiltration into damaged muscle tissue. This delay in the degenerative phase further results in a slowed migration of satellite cells into the necrotic areas and ultimately reducing muscle fiber size in the regenerating muscle of type 1 diabetics. Importantly, our findings demonstrate, for the first time, that muscle group-specific differences exist. Intrinsic differences within muscle groups in the proteolytic cascade (e.g. MMP-9) allow oxidative, postural muscles, such as the soleus, to effectively regenerate despite the negative influence of a systemic rise in PAI-1. Future experiments unravelling the mechanisms affording the oxidative soleus muscle resistance to other aspects of diabetic myopathy (e.g. altered metabolism, impaired growth) will be critical to developing therapeutic strategies aimed at maintaining skeletal muscle health in the face of T1DM.

## Supporting Information

Figure S1 **Macrophage content in regenerating regions of Akita diabetic muscles is not different from wild-type muscles.** In actively regenerating areas of the 5 day post CTX-injured (A) TA, (B) gastrocnemius (GAS) and (C) soleus (SOL) muscles, no significant alteration in macrophage number was found. (D) Representative images of regenerating muscles stained for F4/80 (red) and type I collagen (green). Inset of TA muscles is provided without green channel to clearly demonstrate F4/80 stain morphology. Scale bar represents 50 um.
(TIF)

Figure S2 **Growth of Myh3-positive cells is delayed in regenerating Akita tibialis anterior and gastrocnemius but not soleus muscles.** The average size of Myh3-positive objects was determined and it was found that the TA (A) exhibited a strong trend for a delay in the growth of Myh3-positive cells (P = 0.078), while the delay in growth in the gastrocnemius (GAS) (B) was statistically significant (interaction: P<0.05). The soleus (SOL) exhibited no trend for a delay in growth of Myh3-positive cells (P = 0.42).
(TIF)

Table S1 **Pax7-positive cell density in muscle groups of untreated and PAI-039 treated WT and Akita mice.** TA, gastrocnemius and soleus muscles were analyzed for Pax7-expressing cells in the regenerating, necrotic and undamaged regions of CTX-injected skeletal muscles. Data are presented as mean (±SEM). Untreated group received no treatment, while WT mice in the “Vehicle/PAI-039” group received vehicle and Akita mice received PAI-039 treatment. * denotes groups where n = 2.
(DOCX)
