# Supplementary figures and images for: Impaired Macrophage and Satellite Cell Infiltration Occurs in a Muscle-Specific Fashion Following Injury in Diabetic Skeletal Muscle
Source: PLoS One. 2013 Aug 12;8(8):e70971. doi: 10.1371/journal.pone.0070971 (PMC3741394; doi:10.1371/journal.pone.0070971)

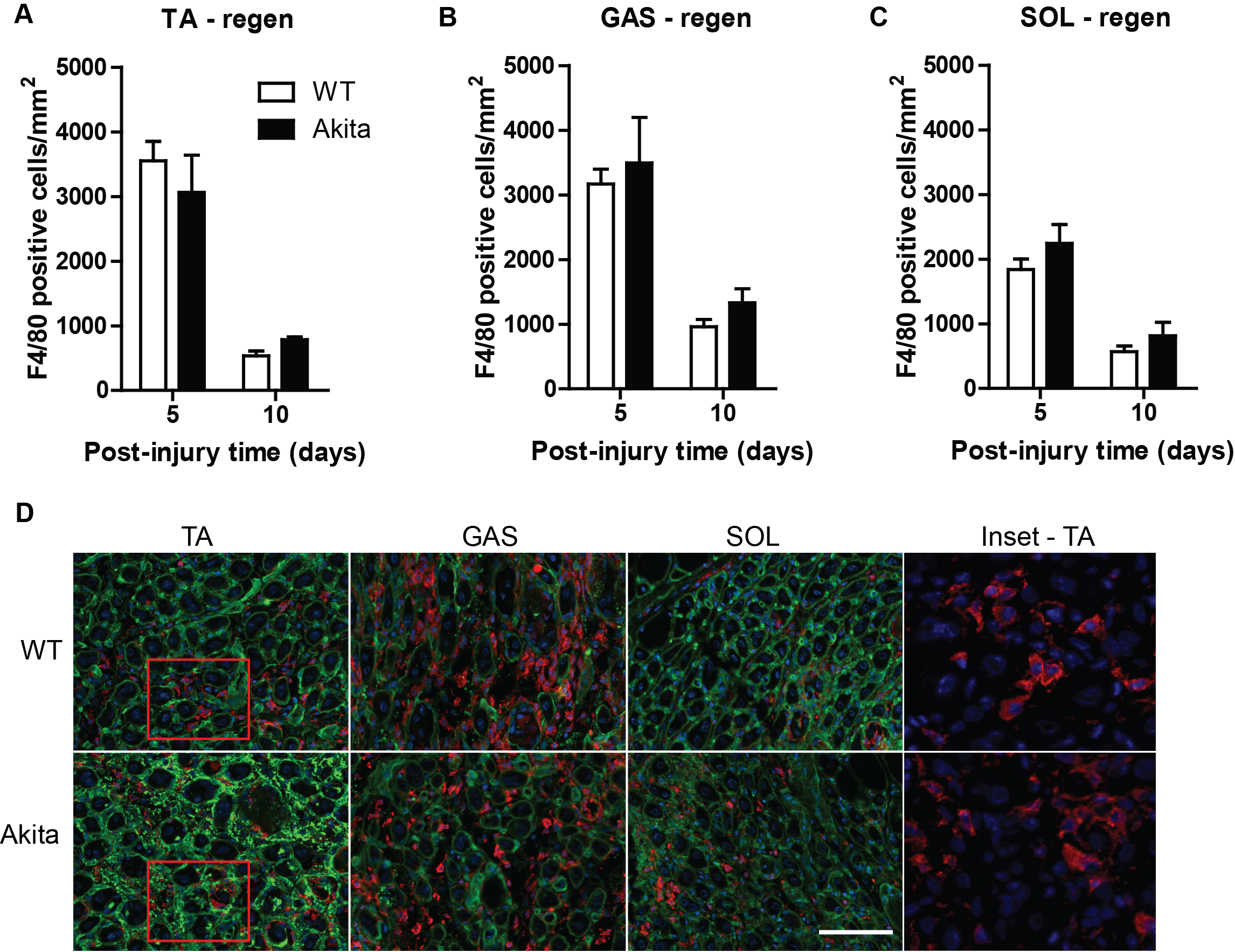

Supplement: Figure S1 — Macrophage content in regenerating regions of Akita diabetic muscles is not different from wild-type muscles. In actively regenerating areas of the 5 day post CTX-injured (A) TA, (B) gastrocnemius (GAS) and (C) soleus (SOL) muscles, no significant alteration in macrophage number was found. (D) Representative images of regenerating muscles stained for F4/80 (red) and type I collagen (green). Inset of TA muscles is provided without green channel to clearly demonstrate F4/80 stain morphology. Scale bar represents 50 um. (TIF) [file pone.0070971.s001.tif]

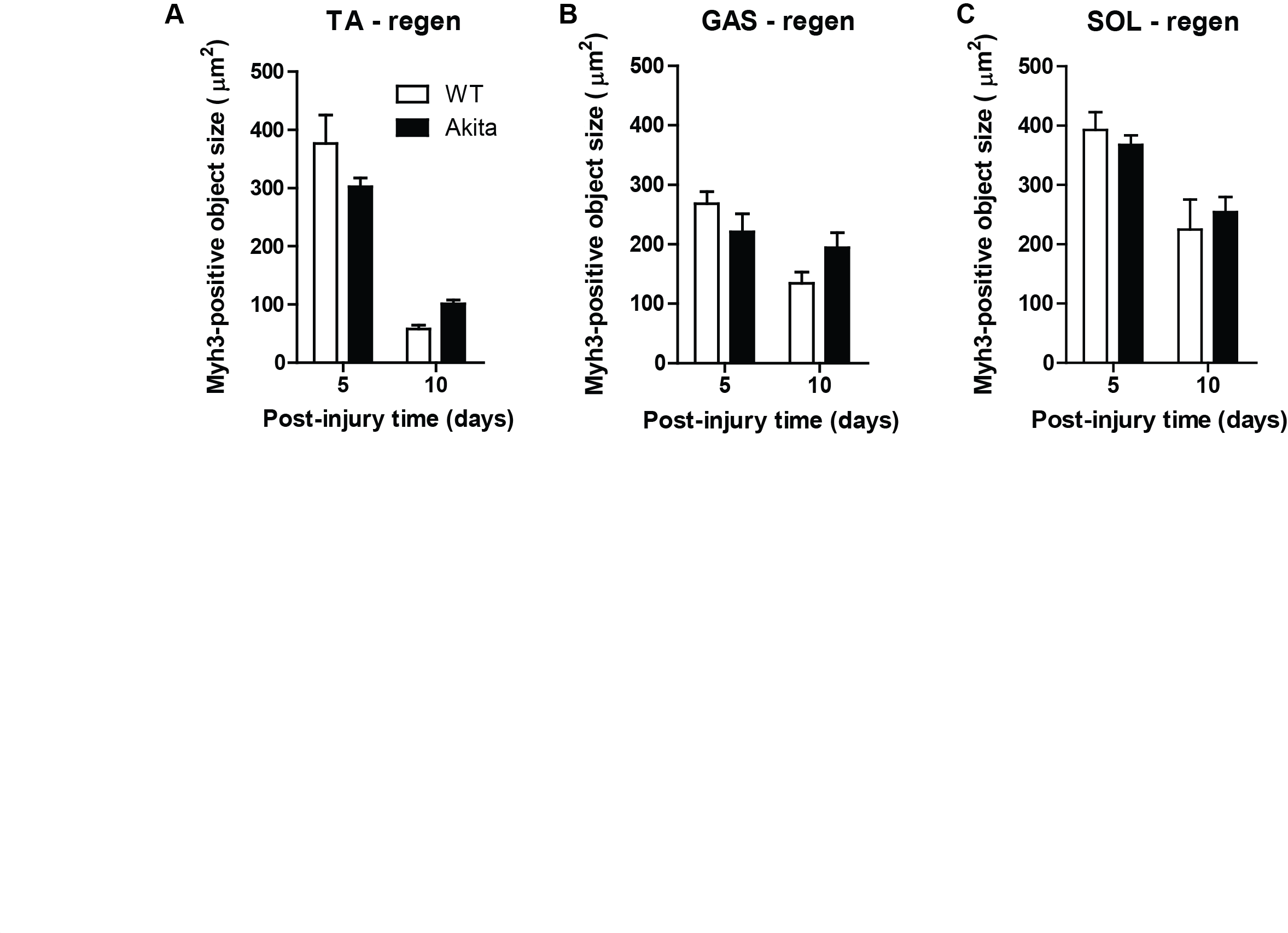

Supplement: Figure S2 — Growth of Myh3-positive cells is delayed in regenerating Akita tibialis anterior and gastrocnemius but not soleus muscles. The average size of Myh3-positive objects was determined and it was found that the TA (A) exhibited a strong trend for a delay in the growth of Myh3-positive cells (P = 0.078), while the delay in growth in the gastrocnemius (GAS) (B) was statistically significant (interaction: P<0.05). The soleus (SOL) exhibited no trend for a delay in growth of Myh3-positive cells (P = 0.42). (TIF) [file pone.0070971.s002.tif]
