# Supplementary material for: Impaired Macrophage and Satellite Cell Infiltration Occurs in a Muscle-Specific Fashion Following Injury in Diabetic Skeletal Muscle
Source: PLoS One. 2013 Aug 12;8(8):e70971. doi: 10.1371/journal.pone.0070971 (PMC3741394; doi:10.1371/journal.pone.0070971)
Supplement: Table S1 — Pax7-positive cell density in muscle groups of untreated and PAI-039 treated WT and Akita mice. TA, gastrocnemius and soleus muscles were analyzed for Pax7-expressing cells in the regenerating, necrotic and undamaged regions of CTX-injected skeletal muscles. Data are presented as mean (±SEM). Untreated group received no treatment, while WT mice in the “Vehicle/PAI-039” group received vehicle and Akita mice received PAI-039 treatment. * denotes groups where n = 2. (DOCX) [file pone.0070971.s003.docx]

|  |  | **Undamaged** | | **Regenerating** | | **Necrotic** | |
| --- | --- | --- | --- | --- | --- | --- | --- |
|  |  | **WT** | **Akita** | **WT** | **Akita** | **WT** | **Akita** |
| **Untreated** | **TA** | 9.8 (2.9)* | 4.0 (0.7)* | 29.8 (7.5) | 22.7 (5.1) | 6.7 (2.1) | 1.4 (0.8) |
|  | **Gastroc** | 13.9 (2.5) | 18.5 (2.9) | 40.2 (8.6) | 43.5 (8.8) | 7.0 (1.4) | 2.7 (0.9) |
|  | **Soleus** | 37.4 (7.4) | 29.9 (2.4) |  |  |  |  |
| **Vehicle/PAI-039** | **TA** | 15.4 (2.5)* | 13.1 (0.6)* | 43.6 (7.9) | 32.3 (2.7) | 10.7 (2.6) | 10.7 (2.8) |
|  | **Gastroc** | 24.2 (1.2) | 17.9 (4.1) | 36.2 (5.4) | 24.4 (7.2) | 12.9 (2.1) | 8.5 (1.8) |
|  | **Soleus** | 44.6 (9.6) | 17.2 (1.8) |  |  |  |  |
